# EARLY DETECTION OF ALZHEIMER’S DISEASE AND RELATED DEMENTIAS FROM VOICE RECORDINGS: THE FRAMINGHAM HEART STUDY

**DOI:** 10.1093/geroni/igad104.3291

**Published:** 2023-12-21

**Authors:** Huitong Ding, Adrian Lister, Cody Karjadi, Rhoda Au, Honghuang Lin, Brian Bischoff, Phillip Hwang

**Affiliations:** Boston University, Chobanian & Avedisian School of Medicine, Boston, Massachusetts, United States; Headwaters Innovation, Inc., Inver Grove Heights, Minnesota, United States; Framingham Heart Study, Framingham, Massachusetts, United States; Boston University, Chobanian & Avedisian School of Medicine, Boston, Massachusetts, United States; UMass Chan Medical School, Boston, Massachusetts, United States; Headwaters Innovation, Inc., Inver Grove Heights, Minnesota, United States; Boston University School of Public Health, Boston, Massachusetts, United States

## Abstract

With the aging global population and the increasing prevalence of dementia, there is a growing focus on identifying mild cognitive impairment (MCI), a pre-dementia state, to enable timely interventions that could potentially slow down neurodegeneration. Producing speech is a cognitively complex task that engages various cognitive domains, while the ease of audio data collection underscores the potential cost-effectiveness and noninvasiveness that voice may offer. This study aims to construct a machine learning pipeline that incorporates speaker diarization, feature extraction, feature selection, and classification, to identify a set of acoustic features that exhibit strong MCI detection capability. The study included 100 MCI cases and 100 healthy controls (HC) matched for age, sex, and education from the Framingham Heart Study. Participants’ speech responses during cognitive tests were recorded, and the recorded audio was processed to identify segments of each participant’s voice from recordings that included voices of both testers and participants. A comprehensive set of 6385 acoustic features was then extracted from these voice segments using the OpenSMILE and Praat softwares. Subsequently, we constructed a random forest model using the features that exhibited significant differences between the MCI and HC groups. We identified an optimal subset of 29 features that resulted in an AUC of 0.87, with a 90% confidence interval ranging from 0.82 to 0.93. This study showcases the potential of human voice as a valuable resource for improving early detection of ADRD and motivates future opportunities to use passive voice collection tools, such as hearing aids, to measure brain health.

